# Genome-centric metagenomics provides insights into the core microbial community and functional profiles of biofloc aquaculture

**DOI:** 10.1128/msystems.00782-24

**Published:** 2024-09-24

**Authors:** Meora Rajeev, Ilsuk Jung, Ilnam Kang, Jang-Cheon Cho

**Affiliations:** 1 Department of Biological Sciences and Bioengineering, Inha University, Incheon, South Korea; 2 Institute for Specialized Teaching and Research, Inha University, Incheon, South Korea; 3 Center for Molecular and Cell Biology, Inha University, Incheon, South Korea; University of Illinois at Chicago, Chicago, Illinois, USA

**Keywords:** biofloc technology, floc-associated microbiota, metagenomics, metagenome-assembled genomes, functional composition, nitrogen cycling

## Abstract

Bioflocs are microbial aggregates that play a pivotal role in shaping animal health, gut microbiota, and water quality in biofloc technology (BFT)-based aquaculture systems. Despite the worldwide application of BFT in aquaculture industries, our comprehension of the community composition and functional potential of the floc-associated microbiota (FAB community; ≥3 µm size fractions) remains rudimentary. Here, we utilized genome-centric metagenomic approach to investigate the FAB community in shrimp aquaculture systems, resulting in the reconstruction of 520 metagenome-assembled genomes (MAGs) spanning both bacterial and archaeal domains. Taxonomic analysis identified *Pseudomonadota* and *Bacteroidota* as core community members, with approximately 93% of recovered MAGs unclassified at the species level, indicating a large uncharacterized phylogenetic diversity hidden in the FAB community. Functional annotation of these MAGs unveiled their complex carbohydrate-degrading potential and involvement in carbon, nitrogen, and sulfur metabolisms. Specifically, genomic evidence supported ammonium assimilation, autotrophic nitrification, denitrification, dissimilatory nitrate reduction to ammonia, thiosulfate oxidation, and sulfide oxidation pathways, suggesting the FAB community’s versatility for both aerobic and anaerobic metabolisms. Conversely, genes associated with heterotrophic nitrification, anaerobic ammonium oxidation, assimilatory nitrate reduction, and sulfate reduction were undetected. Members of *Rhodobacteraceae* emerged as the most abundant and metabolically versatile taxa in this intriguing community. Our MAGs compendium is expected to expand the available genome collection from such underexplored aquaculture environments. By elucidating the microbial community structure and metabolic capabilities, this study provides valuable insights into the key biogeochemical processes occurring in biofloc aquacultures and the major microbial contributors driving these processes.

**IMPORTANCE:**

Biofloc technology has emerged as a sustainable aquaculture approach, utilizing microbial aggregates (bioflocs) to improve water quality and animal health. However, the specific microbial taxa within this intriguing community responsible for these benefits are largely unknown. Compounding this challenge, many bacterial taxa resist laboratory cultivation, hindering taxonomic and genomic analyses. To address these gaps, we employed metagenomic binning approach to recover over 500 microbial genomes from floc-associated microbiota of biofloc aquaculture systems operating in South Korea and China. Through taxonomic and genomic analyses, we deciphered the functional gene content of diverse microbial taxa, shedding light on their potential roles in key biogeochemical processes like nitrogen and sulfur metabolisms. Notably, our findings underscore the taxa-specific contributions of microbes in aquaculture environments, particularly in complex carbon degradation and the removal of toxic substances like ammonia, nitrate, and sulfide.

## INTRODUCTION

Biofloc technology (BFT) has recently garnered considerable attention as an economically viable and environmentally friendly aquaculture approach, revolutionizing traditional practices by harnessing microorganisms to enhance water quality, nutrient recycling, and overall productivity (1). This technique principally relies on balancing higher carbon-to-nitrogen (C/N) ratio, which stimulates the growth of heterotrophic bacteria. The densely proliferating heterotrophic bacteria amalgamate with various other microorganisms and particulate organic matter, ultimately forming flocculated microbial aggregates, commonly referred to as bioflocs (2). These floc-associated microbes, hereinafter referred to as the FAB community, are considered crucial not only for eliminating excessive nutrients, including deleterious inorganic nitrogenous components like ammonia, nitrite, and nitrate but also for serving as a health regulator and supplementary food sources for the growing animals (3).

Considering its importance in BFT, several earlier studies have investigated the microbiota composition of the FAB community. Our recent study uncovered a profound difference in the richness, community composition, and inter-species interactions of the FAB community and peripheral planktonic microbiota (4). Likewise, Wei et al. (5) suggested that biofloc size influences FAB community composition , while Huang et al. (6) found that microbiota in large-sized bioflocs closely resembles shrimp gut microbiota. Conversely, Souza et al. (7) reported no influence of biofloc sizes on the nitrification process. Furthermore, the FAB community has been proven to positively influence the growth, gut microbiota, innate immunity, and disease tolerance of cultured animals, including shrimps and fish (7–10).

While prior research has investigated the microbial members of the FAB community, substantiate gaps remain in understanding the precise community structure, genomic potential, and ecological roles of individual microbial taxa in biofloc aquaculture. Existing studies have primarily relied on metataxonomic approaches, such as 16S rRNA gene amplicon sequencing, which often fail to accurately depict taxonomic composition due to primer mismatch and PCR-induced biases, and do not capture the genome content of microorganisms (11). To the best of authors’ knowledge, a comprehensive study delineating bacterial community composition and functional potential of the FAB community through shotgun metagenomic sequencing coupled with state-of-the-art bioinformatics tools has not yet been conducted. Nonetheless, the demand for utilizing metagenomic approaches in aquaculture has been frequently expressed (4, 12).

Recent advancements in metagenomics have transformed our understanding of the previously unexplored genetic diversity hidden within as-yet-uncultured microorganisms, often referred to as the “microbial dark matter” (13). A pivotal development in this area involves the reconstruction of microbial genomes, known as metagenome-assembled genomes (MAGs), from complex metagenomic data sets using *de novo* assembly and binning algorithms (14). This strategy offers a culture-independent means to elucidate the genomic repertoire of individual microorganisms, providing insights into their metabolic capabilities and potential ecological roles in complex communities (15). The recovery of microbial MAGs has greatly expanded scientific understanding of previously uncharacterized microbial taxonomy and functional guild across various natural environments, including deep-sea sediment and human gastrointestinal tracts (16, 17). Similarly, engineered ecosystems such as wastewater treatment plants (WWTPs) and landfill leachate systems, which leverage microbial activity for pollutant removal akin to biofloc aquaculture, have also undergone extensive studies for MAGs recovery, elucidating microbial dark matter and their functional potential (18–20). Despite the increasing use of metagenomics across various fields, this approach remains underutilized in certain agro-industrial disciplines, including aquaculture.

In this study, we applied a genome-centric metagenomic approach for the first time to investigate the FAB community in biofloc aquaculture systems. Our primary objectives were to unravel the precise taxonomic composition of the floc-associated microbiota and link this microbial structure to their potential metabolic functions. To achieve this, biofloc metagenomes (≥3 µm prokaryotic size fraction) obtained from a commercial aquaculture system in South Korea, as well as publicly available from China (21), were analyzed together. This effort led to the reconstruction of 520 MAGs, and a comprehensive investigation of their associated genes involved in key biogeochemical processes such as carbon, nitrogen, and sulfur cycling.

Understanding the precise composition and genomic potential of the FAB community can advance aquaculture in several ways. First, it promotes sustainable practices through targeted microbial management. By identifying specific microbes and their roles in nitrogen cycling and organic matter decomposition, environmental conditions can be optimized to enhance the conversion of waste into biomass. This reduces the release of harmful substances like ammonia and nitrate, minimizes water exchange, and lowers the risk of pathogen introduction. Second, it improves animal health by modulating their gut microbiota. Identifying beneficial microbial groups, such as probiotics, can lead to the development of microbial inoculants that enhance immunity and disease resistance. For instance, gut microbiota transplantation of a synthetic bacterial consortium comprising *Paracoccus*, *Ruegeria*, *Microbacterium*, *Demequina*, and *Tenacibaculum* from a biofloc system improved shrimp growth, suppressed disease caused by *Vibrio parahaemolyticus*, and restored health in diseased shrimp (22). Additionally, microbiome manipulation can reduce the need for antibiotics and the spread of antibiotic resistance (23).

Overall, a deep understanding of the FAB community will enable precise control over biofloc systems, enhance nutrient recycling, reduce environmental impact, and ultimately improve aquaculture sustainability and productivity.

## MATERIALS AND METHODS

### Biofloc sampling and shotgun metagenomic sequencing

Detailed information about the BFT-based aquaculture system and the samples utilized in this study can be found in our recent publications (4, 24). In brief, the study was conducted at a commercial biofloc aquaculture system located in Ganghwa-do, Incheon, Republic of Korea (37.7000 N, 126.3888 E). This aquaculture system uses molasses to maintain the C/N ratio, thereby fostering the growth of heterotrophic bacteria for biofloc development.

A total of eight rearing water samples were collected at different growth stages of two *Litopenaeus vannamei* batches (Batch-1 and Batch-2) between April 2018 and July 2018 (Table S1). The first five metagenomes represent the FAB community colonized during the growth of Batch-1, while the later three metagenomes represent the FAB community colonized during the growth of Batch-2 (Table S1). Water samples were randomly collected from three different sites in the growth tank and combined to prepare a representative sample. A volume of 1 L water from each representative sample was gently centrifuged to recover high-density bioflocs. The supernatants of resulting rearing water were subsequently filtered through 3 µm pore size membrane filters (Advantech MFS, Inc., Japan) to obtain smaller biofloc particles. These two fractions were finally combined to obtain the FAB community (≥3 µm size fraction) and nucleic acid was extracted using the DNeasy PowerSoil DNA isolation kit (QIAGEN, Hilden, Germany).

The Illumina library preparation and sequencing were conducted following the standard shotgun metagenomic sequencing protocol. In brief, libraries were prepared using the Illumina TruSeq Nano DNA (350) kit based on the manufacturer’s protocol, and 150 bp paired-end (PE) reads were generated on an Illumina HiSeq X platform (Illumina, CA, USA) at Macrogen Inc. (Seoul, Republic of Korea).

### Biofloc metagenomes acquisition from the public repository

As detailed above, eight biofloc metagenomes were collected in this study. Considering the limited availability of microbial genomes from biofloc aquaculture system, we supplemented our data set by downloading three additional biofloc metagenomes from the NCBI’s Sequence Read Archive (SRA) to recover a higher number of MAGs. These additional metagenomes were originally deposited as part of a study conducted by Chen et al. (21), exploring the patterns of antibiotic resistance genes in a biofloc aquaculture system. Accession numbers and other relevant details for all metagenomes utilized in our study for MAGs recovery are provided in Table S1.

### Quality control, assembly, and binning of metagenomic reads

**Fig 1:**
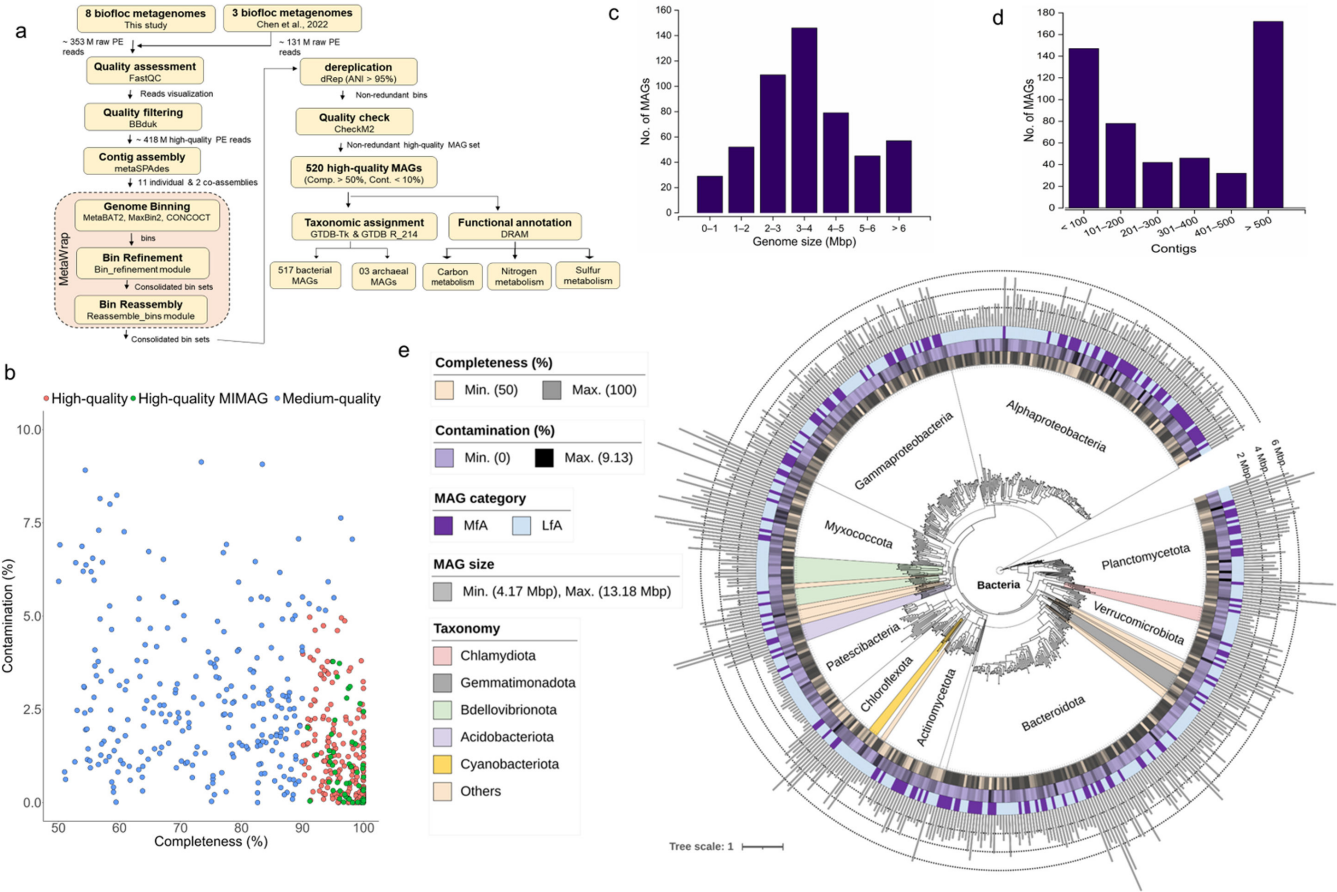
Genome-centric recovery of MAGs from the FAB community. (**a**) Schematic depiction of the bioinformatics workflow followed in this study. (**b**) Distribution of MAGs according to their completeness and contamination levels. (**c and d**) Distribution of retrieved MAGs based on their genome sizes and contigs count. (**e**) Maximum likelihood phylogenetic tree of bacterial MAGs. The tree was constructed in RAxML with 150 bootstraps iterations based on the concatenated alignment of bacterial 122 single-copy marker proteins using GTDB-Tk. Major bacterial phyla are either labeled on the tree or distinguished with different colors. The inner, middle, and upper rings represent MAGs completeness, contamination, and frequency categories, respectively. The outermost bar plots indicate the size of each MAG. The low-abundant bacterial phyla constituted by ≤5 MAGs are indicated as “others.” Detailed assembly statistics of each MAG are provided in Table S3. ANI, average nucleotide identity; LfA, least frequently appeared MAGs; MAGs, metagenome-assembled genomes; MfA, most frequently appeared MAGs; MIMAG, minimum information about MAG.

The bioinformatics workflow implemented in this study is illustrated (Fig. 1a). Metagenomes were initially processed for adapter trimming and contaminant screening using BBDuk v39.01. We removed any reads having Phred quality score <20 and length <100 nucleotides. The quality-filtered reads underwent assembly using metaSPAdes v3.15.4 with the parameters “-k 21,33,55,77,99,127 --meta” (25). Initially, individual assemblies were performed for all metagenomes. Then, metagenomes derived from the same studies were co-assembled, resulting in the generation of two co-assemblies (co-assembly 1 and co-assembly 2). Here, we refrained from performing co-assembly across samples from different studies to avoid the formation of chimeric genomes (26). Our approach involved individual assembly to obtain high-quality bins for abundant bacterial groups, while the co-assembly approach facilitated the recovery of genomes with lower abundance (27, 28).

Contigs (≥1,000 bp length) of each assembly were then used to recover bins using metaWRAP v1.3.2 pipeline (29), which by default employs three binning algorithms, including MetaBAT2, MaxBin2, and CONCOCT. Bins generated from these three algorithms were refined using the Bin_refinement module of metaWRAP (parameters: -c 50, -x 10). The consolidated bin sets from individual and co-assemblies were de-replicated using dRep v3.4.2 (average nucleotide identity; ANI ≥95%) to remove redundant bins and select the best quality ones (27). Finally, a compendium of 520 non-redundant species-level MAGs with medium- to high-quality characteristics (completeness >50%; contamination <10%) was recovered after quality evaluation using CheckM2 v1.0.1 (30). We estimated their quality scores by deducing five times contamination from the completeness (31). Taxonomic assignment was performed using the Genome Taxonomy Database toolkit (GTDB-Tk) v2.3.0, utilizing the latest GTDB release (R08-RS214).

### Metagenomic coverage of MAGs compendium

Abundance estimation was performed by aligning metagenomic reads to the recovered MAGs using CoverM v0.6.1 (https://github.com/wwood/CoverM). In addition to biofloc metagenomes, this analysis included metagenomes from similar environments such as rearing water (*n* = 3), shrimp intestines (*n* = 5), and marine biofilms (*n* = 15) from earlier studies (21, 32, 33) (Table S2). This approach aimed to evaluate the representation of our MAGs collection across bioflocs and other similar environments. Metagenomes from rearing water and shrimp intestines were included because we wanted to assess the representativeness of our MAGs compendium in these two crucial components of the biofloc aquaculture system. The marine biofilm metagenomes utilized here represent microbial communities attached to various artificial surfaces in the coastal seawater of Hong Kong, China (33). This served as an outgroup, representing an attached marine community similar to the FAB community but unrelated to biofloc aquaculture.

For this analysis, all metagenomes underwent quality filtering and were subsampled to 10 million PE reads using Seqtk (https://github.com/lh3/seqtk). On the other hand, sequences corresponding to ribosomal and tRNA genes within each MAG were masked to ensure precise interpretation. Metagenomic reads were then aligned against the recovered MAGs collection using the following CoverM parameters: -m count, -x fa, --min-read-aligned-length 50, --min-read-percent-identity 95, --min-read-aligned-percent 50, and --min-covered-fraction 0. Relative abundance calculations for each MAG were based on the total metagenomic PE reads.

### Functional annotations

Both direct metagenome contigs and recovered MAGs were functionally annotated using the distilled and refined annotation of metabolism (DRAM) pipeline v1.4.6 (34) with default settings. Initially, a filtering step was implemented to remove short-length contigs (>2,500 bp), followed by the identification of open reading frames (ORFs) using Prodigal. Predicted protein coding sequences (CDSs) were then queried against multiple databases, including KOfam, UniRef90, Pfam, CAZY, and MEROPS, using the *DRAM.py annotate* function. A summarized view of the crucial metabolic pathways was obtained using the *DRAM.py distill* function of DRAM.

### Identifying specific metabolic pathways through marker genes

The potential occurrence of heterotrophic nitrification-aerobic denitrification (HN-AD) in biofloc aquaculture systems has long been a subject of interest (35). To investigate the possibility of this process, we conducted a BLASTp analysis aimed at identifying key marker enzymes associated with HN-AD, specifically dinitrogen-forming (DnfABC) and pyruvic oxime dioxygenase (POD) (36). The analysis involved enzymes DnfABC (GenBank protein identifiers QXX79842–QXX79844) and POD (GenBank protein identifier GAU72725) as query sequences against all recovered MAGs in our study, along with the genomes of two reference species: *Alcaligenes ammonioxydans* strain HO-1 (GenBank genome accession CP049362) and *Alcaligenes faecalis* strain JQ135 (GenBank genome accession CP021641). These reference species are known for their capability to perform HN-AD (36–38).

Also, five MAGs were identified to harbor dissimilatory sulfite reductases (*dsrAB*) genes in DRAM analysis. Bacterial *dsrAB* can exist in two forms: (i) reductive *dsrAB*, which catalyze sulfite to sulfide reduction during anaerobic respiration in sulfur-reducing bacteria, and (ii) reverse/oxidative *dsrAB* (*rdsrAB*), acting in reverse order to oxidize sulfide to sulfite in sulfur-oxidizing bacteria (39). The type of *dsrAB* in the recovered MAGs was confirmed by determining their phylogenetic placement in the *dsrAB* genes reference tree (40).

### Phylogenetic trees construction

The phylogenetic trees of bacterial and archaeal MAGs were constructed based on 120 bacterial- and 53 archaeal-specific single-copy marker protein alignments by the GTDB-Tk toolkit. Bacterial tree was inferred using RAxML v8.2.12 (options: -f a -# autoMRE -m PROTGAMMAAUTO), while archaeal tree using FastTree (options: -lg -gamma). Bacterial 16S rRNA gene sequences were extracted from recovered MAGs using Bedtools and sequences with >1,000 nucleotide length were compared against the EzBioCloud database to identify novel taxa. Next, all 16S rRNA gene sequences were aligned using ClustalW program in MEGAX software, and phylogenetic tree was obtained using RAxML (options: -f a -# 1000m GTRGAMMA). All trees were finally annotated and decorated in Interactive Tree of Life (iToL) v6.8.

### Accessibility to meta(genome) data sets

The biofloc metagenomes obtained in this study are deposited in the NCBI’s SRA under accession numbers SRR24442552–SRR24442559. Similarly, FASTA files of all the reconstructed MAGs are publicly accessible through FigShare repository (https://doi.org/10.6084/m9.figshare.25249096).

## RESULTS

### Reconstruction of 520 MAGs from the FAB community

The entire methodological roadmap followed in this study is depicted (Fig. 1a). Illumina shotgun sequencing of the eight biofloc metagenomes in this study generated 353.19 million raw PE reads (Table S1). To broaden the data set size and enhance the recovery of MAGs, we included an additional 131.25 million PE reads representing three biofloc metagenomes obtained from the SRA database. Comprehensive Illumina read statistics for the total 11 biofloc metagenomes used in this study are detailed in Table S1. Genome-centric metagenomic analysis utilizing a mix-assembly approach, combining both individual and co-assembly methods, resulted in the recovery of 517 non-redundant bacterial and three archaeal MAGs. Assembly statistics of these species-level MAGs (ANI ≥95%), including completeness, contamination, genome size, quality scores, number of contigs, and GC content, are provided (Tables S3 and S4).

Figure 1 summarizes key characteristics of the reconstructed MAGs affiliated with the bacterial domain. Of the 517 MAGs, 257 were categorized as high-quality (≥90% completeness, ≤5% contamination) and 260 as medium-quality (≥50% completeness, ≤10% contamination) (Fig. 1b; Table S3). Notably, 56 high-quality MAGs possessed all three ribosomal RNA genes (5S, 16S, and 23S) along with more than 18 tRNA genes, meeting the minimum information about a MAG (MIMAG) standard. Bacterial genome sizes varied from 0.14 to 13.18 Mbp, with the majority falling in the range of 2–5 Mbp (Fig. 1c). About half of the MAGs (*n* = 267) contained less than 300 contigs (Fig. 1d).

For the archaeal domain, the recovered MAGs were affiliated with the phyla *Thermoproteota* (two MAGs) and *Nanoarchaeota* (one MAG) (Table S4). The phylogenetic relationship of these archaeal MAGs with their nearest relatives is illustrated (Fig. S1).

### *Rhodobacteraceae* and *Flavobacteriaceae* dominate the FAB community

Taxonomic classification distributed recovered bacterial MAGs across 13 bacterial phyla, including *Pseudomonadota* (formerly *Proteobacteria*; 184 MAGs), *Bacteroidota* (99), *Planctomycetota* (45), *Myxococcota* (34), *Patescibacteria* (29), *Actinomycetota* (25), *Bdellovibrionota* (18), *Verrucomicrobiota* (17), *Chloroflexota* (16), *Acidobacteriota* (7), *Gemmatimonadota* (7), *Chlamydiota* (6), and *Cyanobacteriota* (6) (Fig. 1e; Table S3). Additionally, several low-abundant bacterial phyla were recovered, each represented by fewer than five MAGs, including *Armatimonadota*, ARS69, *Calditrichota*, CLD3, *Delongbacteria*, *Dependentiae*, *Desulfobacterota*, and *Eisenbacteria*.

**Fig 2:**
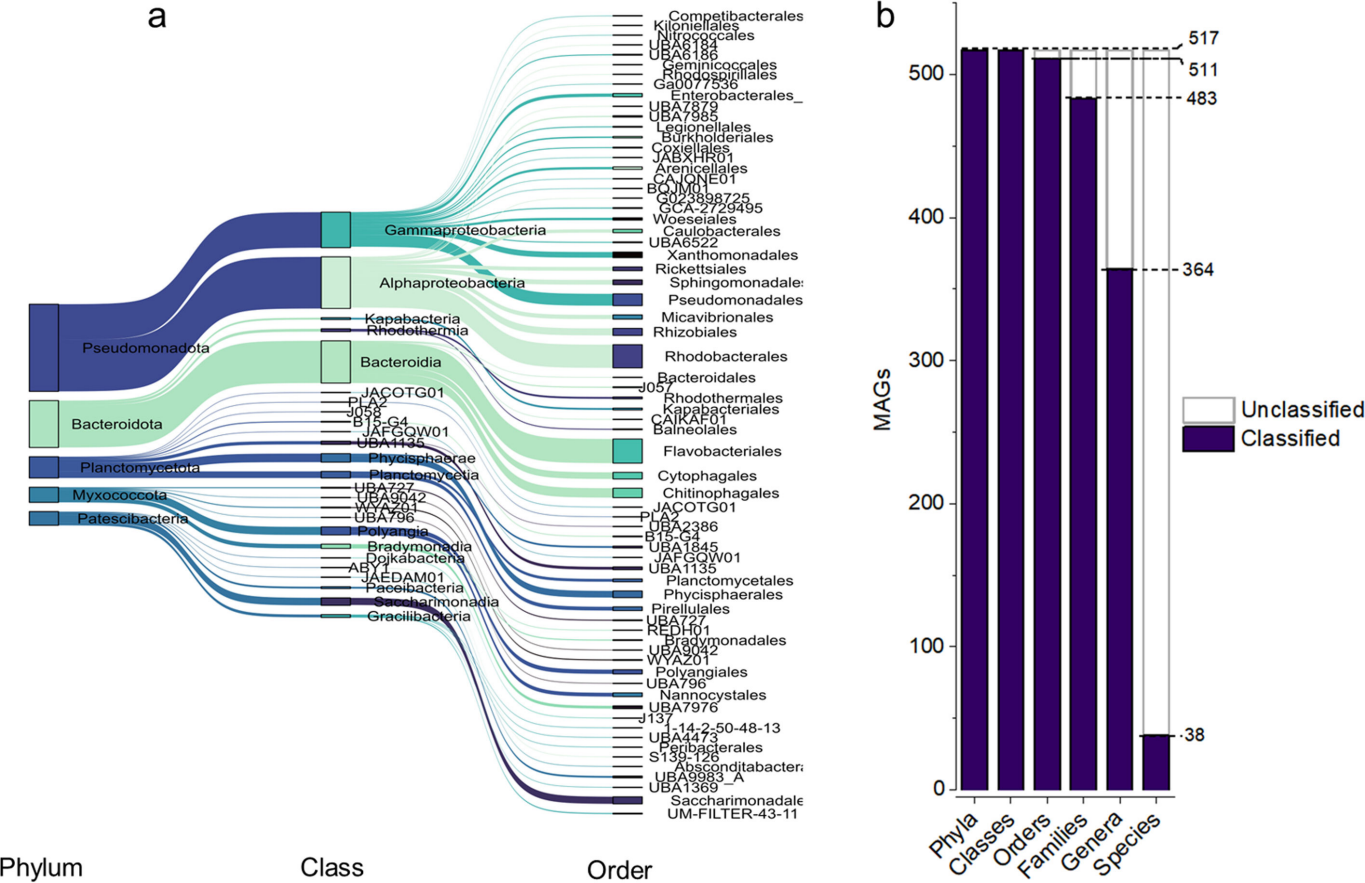
Taxonomic classification of recovered MAGs collection. (**a**) The Sankey plot illustrates the classification of MAGs at three taxonomic levels (phylum, class, and order). For lucidity, MAGs associated with the top five dominant phyla are only displayed. Different colors represent various taxonomic groups, while the height of each node and width of each flow correspond to the number of MAGs associated with respective taxonomic group. (**b**) Bar plots depict the number of classified and unclassified MAGs at various taxonomic ranks based on the GTDB (release R08-RS214).

The principal constituents of the FAB community are depicted in a Sankey plot (Fig. 2a). The highest recovered MAGs were affiliated to the class *Alphaproteobacteria* (109 MAGs), followed by *Bacteroidia* (89 MAGs). Within *Alphaproteobacteria*, the most predominant families were *Rhodobacteraceae* (49 MAGs), *Micavibrionaceae* (9 MAGs) and *Sphingomonadaceae* (7 MAGs) (Table S3). Similarly, *Bacteroidia* were majorly represented by *Flavobacteriaceae* (37 MAGs), *Saprospiraceae* (13 MAGs) and *Cyclobacteriaceae* (8 MAGs). Class *Gammaproteobacteria* (75 MAGs) were represented as the third dominant taxon, with *Woeseiaceae* and *Halieaceae* being the dominant families.

### Prevalence of novel phylogenetic diversity

The distribution of classified and unclassified MAGs across various taxonomic ranks is depicted as bar plots (Fig. 2b). According to the latest GTDB (release R08-RS214), our MAGs collection encompasses six new bacterial orders and 34 new families (Fig. 2b; Table S3). A large proportion of taxa, approximately 30% (153 MAGs), could not be classified at the genus level, with an even more substantial portion (about 93%; 479 MAGs) eluding classification at the species level. This underscores the recovery of several novel genomes in our study and highlights the importance of exploring aquaculture environments for novel microbial phylogeny.

To further substantiate this observation, we extracted nearly full-length 16S rRNA gene sequences from the pool of 129 MAGs. Consistent with the GTDB taxonomy, classification of these 16S rRNA genes revealed several novel bacterial groups compared to currently existing sequences in the EzBioCloud database (Table S5). Among the 129 MAGs, 65 represented hitherto unidentified genera (16S rRNA gene similarity <94.5%), while 48 MAGs exhibited novelties at the species level (16S rRNA gene similarity <98.7%) (Fig. S2).

### Reconstructed MAGs collection is specific and true representative of the FAB community

To determine the representativeness of the obtained MAGs compendium, randomly selected biofloc metagenomic reads were mapped against recovered MAGs. This analysis was also extended to publicly available metagenomes from similar environments, including rearing water and shrimp intestines of biofloc aquacultures, as well as marine biofilms. Relative abundances of each MAG in the investigated metagenomes are detailed in Table S6. As expected, the recovered MAGs exhibited the highest reads mapping (average >59%) with biofloc metagenomes (Fig. S3a and b; Table S7), signifying the robust coverage of our MAGs compendium and the efficacy of the adopted bioinformatics workflow. Subsequent high reads mapping rates were observed in rearing water (averaging 22.97%) and shrimp intestines (averaging 8.21%). In contrast, marine biofilms showed an average mapping of merely <1%. These observations underscore the specificity of recovered MAGs collection to biofloc aquaculture, particularly in the FAB community.

The core bacterial members of the FAB community were determined by categorizing the entire MAGs collection into two groups based on detection frequencies: most frequently appeared (MfA) MAGs, present in ≥6 biofloc metagenomes (>50% of total metagenomes), and least frequently appeared (LfA) MAGs, observed in ≤5 biofloc metagenomes (Fig. S4; Table S8). The MfA category, comprising 184 MAGs with an average relative abundance >0.01%, was considered core member. This analysis suggests that the core microbiota of the FAB community predominantly consist of five bacterial phyla (*Pseudomonadota*, *Bacteroidota, Planctomycetota*, *Actinomycetota*, and *Myxococcota*) and twelve families (*Rhodobacteraceae*, *Halieaceae*, HTCC2089, *Flavobacteriaceae*, *Saprospiraceae*, *Cyclobacteriaceae*, *Planctomycetaceae*, UBA1924, *Ilumatobacteraceae*, *Microbacteriaceae*, *Nannocystaceae*, and UBA1532). Notably, certain genera from *Rhodobacteraceae* (e.g., *Marivita*, *Ruegeria*, *Dinoroseobacter*, *Aliiroseovarius*, *Arenibacterium*, *Dinoroseobacter*, and *Albidovulum*), *Flavobacteriaceae* (e.g., *Muricauda*, JAIOUW01), *Saprospiraceae* (e.g., *Phaeodactylibacter*), and *Microbacteriaceae* (e.g., *Microbacterium*), were consistently present across all investigated biofloc metagenomes (Table S8), suggesting they are the most consistent and core bacterial members of the FAB community.

### Metabolic potential of the FAB community

**Fig 3:**
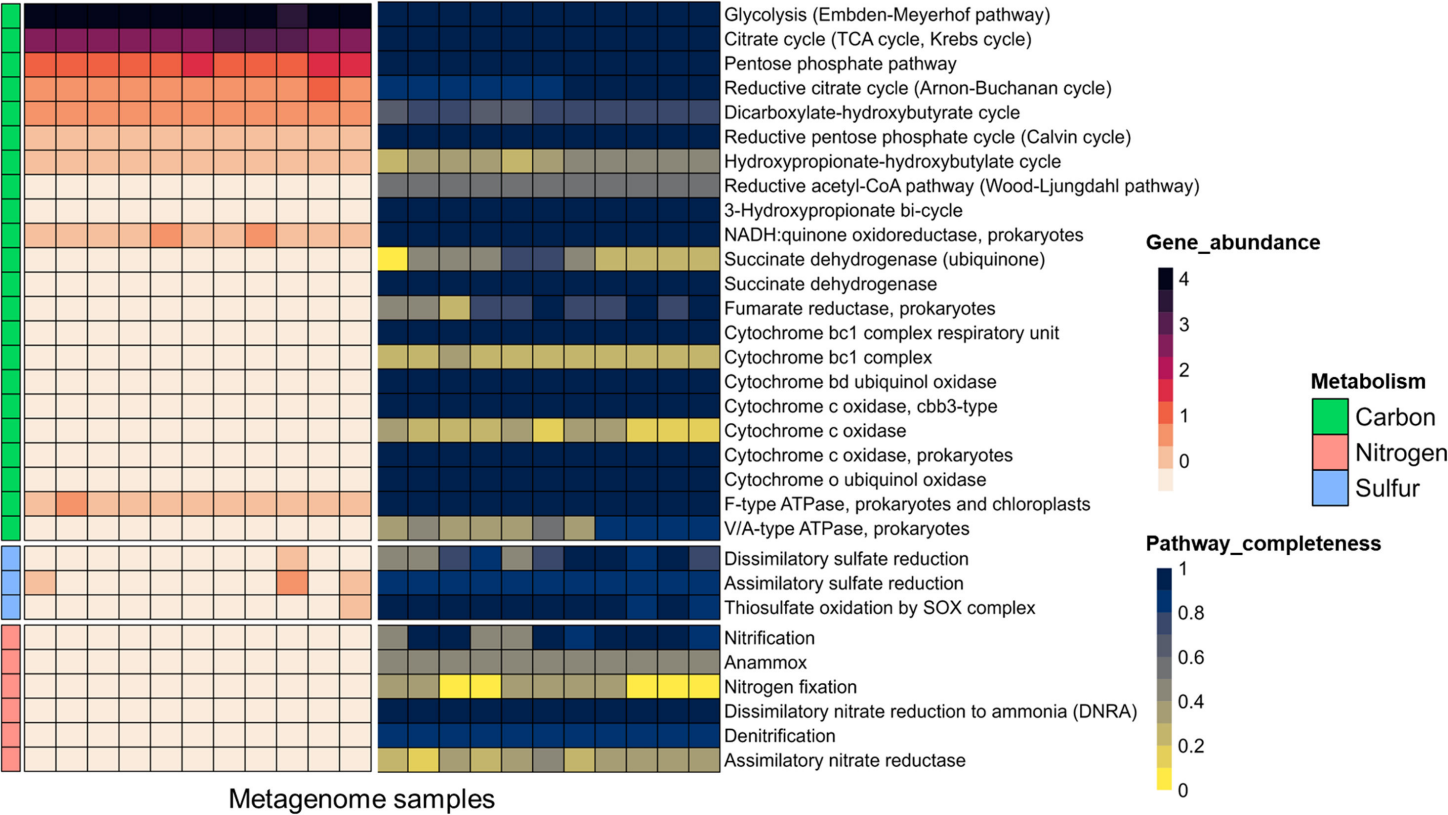
Overall functional potential of the floc-associated bacterial community. Heatmap (left panel) illustrating the gene abundance related to crucial metabolic pathways, including carbon, nitrogen, and sulfur metabolisms, across biofloc metagenomes. Gene counts were first summed for each pathway and then normalized using the z-score method. Heatmap (right panel) shows the completeness of corresponding pathways. This figure is based on the annotation of metagenome contigs using distilled and refined annotation of metabolisms (DRAM) tool. The pathway completeness is determined by the DRAM annotation, as well as manual calculation based on the presence/absence metrics of genes related to a specific module.

We initially conducted annotations on direct metagenome contigs to elucidate the overall functional potential of the FAB community. The abundance of functional genes associated with crucial metabolic pathways (carbon, nitrogen, and sulfur metabolisms) and their corresponding completeness in the metagenome contigs are depicted in Fig. 3. The highest proportion of annotated genes with full completeness were related to central carbon metabolism such as glycolysis, citrate cycle, and pentose phosphate pathways.

In addition to the metagenome contigs, contribution of various taxonomic groups in pivotal metabolic pathway was inferred by annotating recovered MAGs. The genes involved in various metabolic pathways across annotated MAGs are provided in Table S9.

### FAB community encodes numerous carbohydrate-active enzymes (CAZymes)

Considering the copiotrophic conditions in biofloc aquaculture, we assessed the polysaccharide-degrading potential of the FAB community. Annotation of bacterial MAGs identified 7,540 CAZyme-encoding genes distributed across six major classes. The distribution of these CAZyme classes and their corresponding bacterial sources is depicted (Fig. S5a; Table S10). The FAB community majorly encodes two classes of catabolic CAZymes: glycoside hydrolases (GHs) and polysaccharide lyases (PLs), both of which are involved in polysaccharide degradation. The GHs were predominantly occupied by families GH13 (alpha-amylase), GH43 (beta-xylosidase), GH16 (xyloglucosyltransferase), and GH5 (cellulase), which are involved in the degradation of starch, cellulose, and hemicellulose. These genes were primarily encoded by *Bacteroidota* (mostly *Flavobacteriaceae*, *Saprospiraceae* and *Cyclobacteriaceae* members), followed by *Pseudomonadota*, *Planctomycetota*, and *Verrucomicrobiota* (Fig. S5b; Table S10), suggesting the crucial function of these bacterial taxa in complex organic matter degradation and utilization. The abundance of PLs was relatively lower, mostly represented by families PL1 (pectate lyase), PL9 (pectate lyase), PL10 (polysaccharide lyases), and PL12 (heparin-sulfate lyase), with *Bacteroidota*, *Planctomycetota*, and *Verrucomicrobiota* members being the major carriers.

### Genomic evidence supports nitrogen metabolism by the FAB community

**Fig 4:**
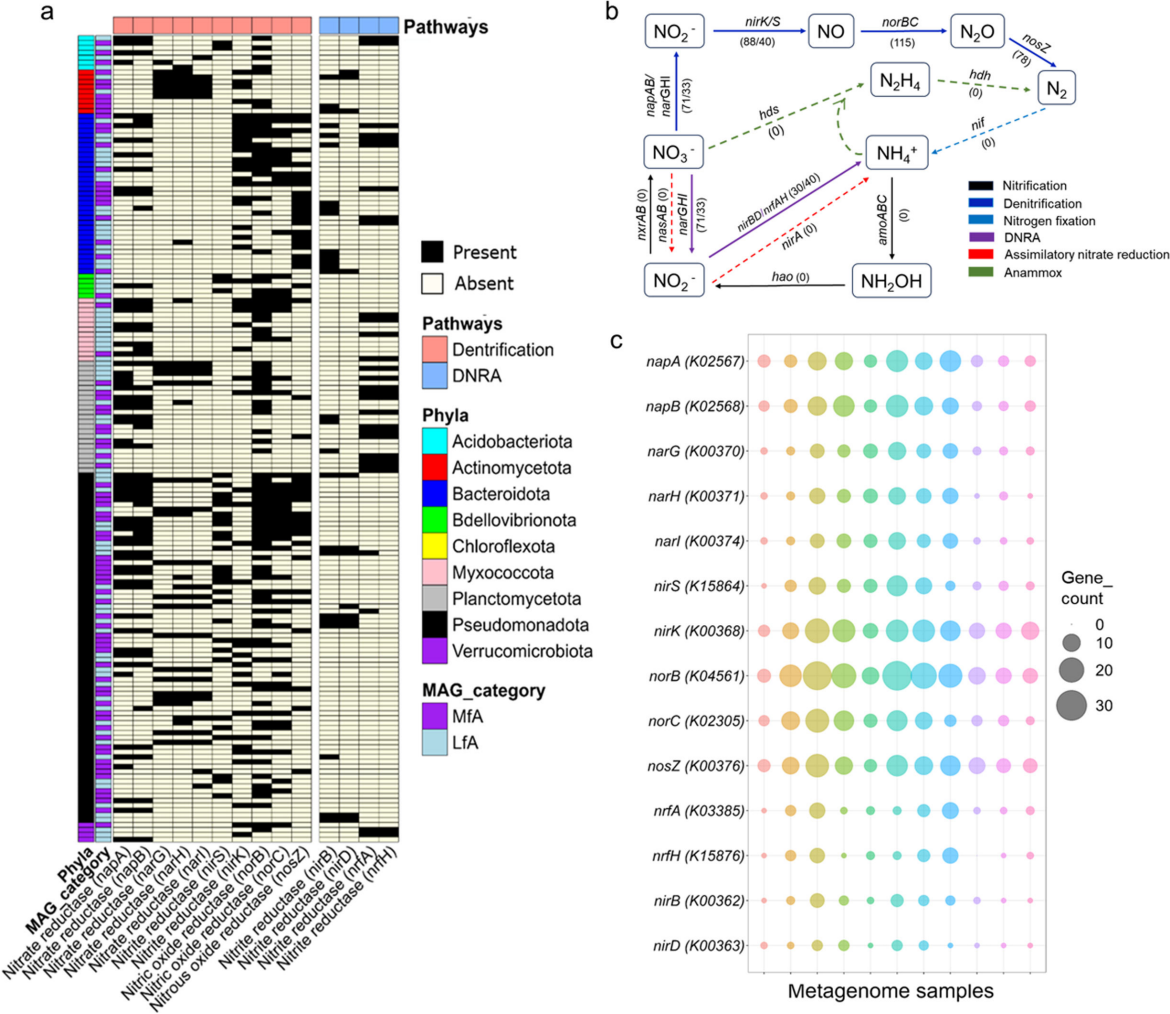
Genomic evidence for nitrogen metabolism by the FAB community. (**a**) Distribution of genes associated with denitrification and DNRA among the recovered MAGs based on their taxonomy. (**b**) Major nitrogen metabolism pathways and the number of MAGs from the FAB community containing genes required for each step. Solid lines indicate pathway presence, while dashed lines denote pathway absence in the FAB community. Numerical values in parentheses denote the number of MAGs harboring corresponding genes. (**c**) Bubble plot represents number of annotated genes related to denitrification and DNRA in the metagenome contigs ofthe investigated biofloc samples. The numerical numbers in parentheses represent KEGG identifiers for each gene. *amoABC*, ammonia monooxygenases; DNRA, dissimilatory nitrate reduction to ammonia; *hao*, hydroxylamine oxidase; *hdh*, hydrazine dehydrogenase; *hzs*, hydrazine synthase; LfA, least frequently appeared MAGs; MfA, most frequently appeared MAGs; *napAB*, periplasmic nitrate reductases; *narGHI*, membrane-bound nitrate reductases; *nas*, assimilatory nitrate reductase; *nif*, nitrogen fixation proteins; *nirA*, ferredoxin-nitrite reductase; *nirBD*, nitrite reductases (NADH); *nirSK*, nitrite reductases (NO-forming); *nor*, nitric oxide reductase; *nos*, nitrous-oxide reductase; *nrfAH*, nitrite reductases (cytochrome); *nxr*, nitrite oxidoreductase.

We first assessed the abundance of genes related to various common nitrogen metabolism processes, including autotrophic nitrification, nitrogen fixation, anaerobic ammonium oxidation (anammox), assimilatory nitrate reduction, denitrification, and dissimilatory nitrate reduction to ammonia (DNRA). The distribution of these genes in metagenome contigs and recovered MAGs is depicted (Fig. 3; Table S11). Both data clearly indicate that the FAB community possesses a higher potential for denitrification (nitrate reduction to dinitrogen) and DNRA (nitrate reduction to ammonia). A complete set of genes required for denitrification (*napAB*, *narGHI*, *nirSK*, *norBC*, *nosZ*) and DNRA (*nirBD*, *nrfAH*) were observed across diverse taxonomic groups (Fig. 4a). The functional marker genes for denitrification (nitrite reductases; *nirS* and *nirK*) and DNRA (nitrite reductases; *nirBD*) were identified in 128 and 30 MAGs, respectively (Fig. 4b and c). Bacterial taxa recognized for their denitrification capabilities were predominantly affiliated with the *Pseudomonadota* (mostly *Rhodobacteraceae* of *Alphaproteobacteria* and *Woeseiaceae* of *Gammaproteobacteria*), and *Bacteroidota* (mostly *Flavobacteriaceae*). DNRA genes are primarily found in *Bacteroidota* (mainly *Saprospiraceae*, *Cyclobacteriaceae*) and *Pseudomonadota* (mainly *Halieaceae* and *Alteromonadaceae*) (Table S11). Additionally, the majority of MAGs associated with denitrification and DNRA are categorized as MfA, suggesting a widespread occurrence of these processes in biofloc aquaculture.

There was minor evidence of autotrophic nitrification, indicated by a low number of ammonia monooxygenase (*amoABC*) genes observed in metagenome contigs, but not in any recovered MAGs (Fig. 3; Fig. 4b). This prompted us to investigate the potential occurrence of heterotrophic nitrifiers. We conducted BLASTp analyses on two key functional marker enzymes, DnfABC and POD, associated with HN-AD, against all recovered MAGs. Heatmaps illustrate differences in the similarity of these proteins between two reference bacterial strains and the top five MAGs exhibiting the highest similarities (Fig. S6a and b). Noticeably, our MAGs collection displayed discernible differences compared to reference bacterial strains, implying a probable lack of heterotrophic nitrifiers in the FAB community.

We then investigated the ammonium assimilation potential of the FAB community. Although it is well-known that BFT principally relies on ammonium assimilation rather than nitrification, the major bacterial members performing this process remain unidentified. Therefore, we identified MAGs potentially involved in two ammonium assimilation pathways: glutamine synthetase/glutamate synthase (GS-GOGAT) and glutamate dehydrogenase (GDH). This analysis suggests that the FAB community prefers the GS-GOGAT pathway over GDH for ammonium assimilation (Fig. S7; Table S12). Genes encoding a complete GS-GOGAT pathway (*glnA*, *gltB*, and *gltD*) were identified in 145 MAGs, mostly categorized as MfA and affiliated with predominant taxonomic groups of the FAB community such as *Cyclobacteriaceae*, *Flavobacteriaceae*, *Halieaceae*, *Rhodobacteraceae*, *Saprospiraceae*, *Sphingomonadaceae*, and *Woeseiaceae*. Genes involved in the GDH pathway were detected in 49 MAGs belonging to *Cyclobacteriaceae*, *Flavobacteriaceae*, *Nannocystaceae*, *Rhodobacteraceae*, and *Halieaceae*.

### Specific bacterial groups possess sulfur-oxidizing potential

**Fig 5:**
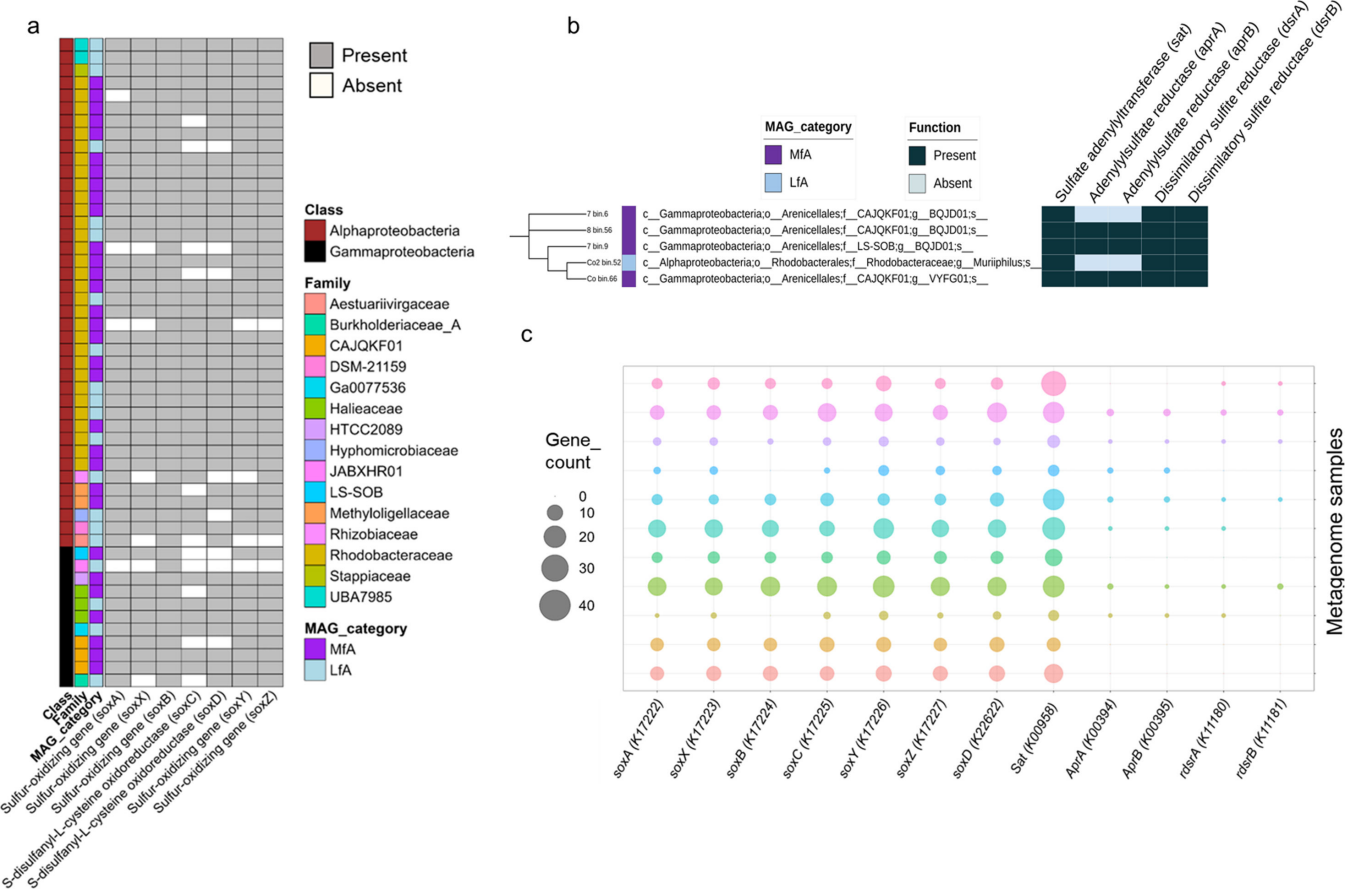
Sulfur metabolic potential of the FAB community. (**a**) Heatmap illustrating the MAGs containing complete *soxXAYZBCD* gens cluster according to their taxonomy. MAGs harboring the *soxB* gene, a molecular marker for *sox*-mediated sulfur oxidation, were included in the heatmap. (**b**) Heatmap illustrating the taxonomic information of MAGs that exhibit both *sox*-mediated and *rdsr*-mediated sulfur oxidation potential. (**c**) Bubble plot depicting the number of genes associated with sulfur oxidation in the metagenome contigs of the investigated biofloc samples. The numerical values in parentheses represent KEGG identifiers for each gene. *apr*, adenylylsulfate reductases; *rdsr*, reverse/oxidative dissimilatory sulfite reductases; *sat*, sulfate adenylyltransferase; *sox*, sulfur-oxidizing genes.

Among the three major sulfur metabolic pathways examined, sulfur oxidation was dominant in the FAB community (Fig. 3; Table S13). About 10% (51 MAGs) harbored a complete sulfur-oxidizing gene complex (*soxXAYZBCD*), suggesting their involvement in oxidizing reduced sulfur species such as sulfite, sulfide, and thiosulfate to sulfate (Fig. 5a; Table S14). All these MAGs contained *soxB* gene, a molecular marker for sulfur-oxidizing bacteria. Further examination identified five MAGs with both *sox* and reverse dissimilatory sulfite reductase (*rdsrAB*) genes (Fig. 5b; Table S15), suggesting their participation also in the reverse sulfate reduction pathway, oxidizing sulfide into sulfate. The oxidative nature of the identified *dsrAB* genes was confirmed through phylogenetic analysis (Fig. S8). The prevalence of genes involved in both *sox-*mediated and *rdsr-*mediated sulfur oxidation across the investigated metagenome contigs is depicted (Fig. 5c), indicating a dominant *sox*-mediated sulfur oxidation potential in the FAB community. Notably, both sulfur-oxidizing pathways were exclusively associated with *Alpha-proteobacteria* and *Gamm-proteobacteria*. The primary *sox*-mediated sulfur oxidizers were members of *Rhodobacteraceae* (mainly genera *Marivita*, *Donghicola*, and *Albidovulum*), while the co-occurrence of both *sox*-mediated and *rdsr*-mediated pathways were primarily associated with the order *Arenicellales*.

## DISCUSSION

This study investigated the microbial composition and metabolic potential of the FAB community, a type of microbial aggregate in biofloc aquaculture systems. We recovered 520 MAGs, with over 90% unclassified at the species level (Fig. 2; Table S3), highlighting the scarcity of cultured representatives and the necessity of employing metagenomics and culturomics approaches in underexplored aquaculture environments. The recovered MAGs exhibited higher read mapping rates even to shrimp intestinal metagenomes (Fig. S3; Table S7), implying a close resemblance of the FAB community to shrimp gut microbiota, potentially impacting shrimp health and immunity. The observed similarity between the intestinal microbiota of growing animals and the FAB community likely arises from their dietary reliance on bioflocs (41).

Given the limited understanding of core microbiota in biofloc aquacultures, we began by understanding the core members of the FAB community. Identifying core microorganisms in complex microbial communities is pivotal, with several methods available for this purpose, including assessing abundance, frequency, inter-species interactions, and functional roles (20). We identified *Rhodobacteraceae*, *Halieaceae*, *Flavobacteriaceae*, *Saprospiraceae*, *Cyclobacteriaceae*, *Planctomycetaceae*, *Microbacteriaceae*, and *Nannocystaceae*, as dominant core members (Fig. 2a; Fig. S4; Table S8). The prominence of these taxa aligns with earlier amplicon-based studies (4, 5). Specifically, core families such as *Rhodobacteraceae*, *Microbacteriaceae*, and *Flavobacteriaceae* have previously been linked to the higher carbon content in biofloc aquacultures (42, 43). Furthermore, these three bacterial groups resist the pathogenic *Vibrio* colonization in shrimp gut by improving gut microbiota stability (22). The dominant core genera include *Marivita*, *Ruegeria*, *Dinoroseobacter*, *Arenibacterium*, *Albidovulum*, *Muricauda*, *Phaeodactylibacter*, and *Microbacterium*, indicating their crucial roles in organic matter degradation, nutrient cycling, and possibly disease suppression. Regular monitoring and optimized aquaculture practices that support the growth of these core genera could improve the overall performance of biofloc aquaculture.

*Rhodobacteraceae* was identified as the most dominant and core member of the FAB community (Fig. 2a; Fig. S4; Table S8). This finding is consistent with previous studies identifying *Rhodobacteraceae* as keystone taxa in rearing water (5) and shrimp intestinal microbiota (6), highlighting the critical role of this taxon in biofloc aquaculture. Furthermore, they are frequently recognized as pioneers and dominant members in similar environments, such as marine biofilms and microbial mats (44), reflecting their surface-colonizing characteristics. The niche-specific success of *Rhodobacteraceae* in the attached marine community may be attributed to several factors: the production of diverse bioactive secondary metabolites that confer competitive advantages (45), a sophisticated quorum sensing system that facilitates community coordination (46), and robust metabolic versatility that enable various energy acquisition mechanisms, including chemolithoautotrophy, photoheterotrophy, and heterotrophy (47, 48).

These characteristics suggest that *Rhodobacteraceae* members could serve as effective probiotics by promoting beneficial microbial interactions and inhibiting pathogens through competitive exclusion and production of antimicrobial compounds. Certain *Rhodobacteraceae* genera such as *Roseobacter* and *Phaeobacter* have already been utilized as safe probiotics in aquaculture (49, 50). Additionally, several *Ruegeria* species (e.g., *R. arenilitoris* and *R. conchae*) have been identified as potential probiotics (43). Strains of *R. mobilis* produce the broad-spectrum antibiotic tropodithietic acid (TDA), confirming their probiotic potential against pathogens (45). A recent study by Guo et al. (22) demonstrated that gut microbiota transplantation involving two *Rhodobacteraceae* genera (*Paracoccus* and *Ruegeria*) resulted in a stable and resistant gut microbiota against pathogenic *Vibrio* infection. Overall, our findings support leveraging *Rhodobacteraceae* as probiotics to improve the resilience and health of aquaculture systems, warranting further investigation.

Next, we investigated the biosynthesis potential of CAZymes and key metabolic pathways, such as nitrogen and sulfur metabolisms, supported by the FAB community. This community predominantly encoded two catabolic CAZymes: GHs (including GH13, GH43, GH16, and GH5), and PLs (specifically PL1) (Fig. S5a and b). Given the prevalent use of starch and cellulose as primary carbon sources in biofloc systems (51), the dominance of genes encoding GHs, responsible for cleaving O-glycosidic bonds in carbohydrates, including alpha-amylases/sucrose phosphorylases, arabino/xylosidases, xyloglucanases, and cellulases, could facilitate the degradation and utilization of these complex polysaccharides. Furthermore, since bioflocs are aggregates of microbes, algae, protozoa, detritus, and dead organic particles, genes encoding cellulases and PL1 pectate lyases could aid in their cell wall degradation.

The phylum *Bacteroidota*, particularly *Flavobacteriaceae* and *Saprospiraceae*, exhibited the highest CAZymes biosynthesis potential (Fig. S5b; Table S10), suggesting their primary role in the degradation of complex organic carbon. These taxa have been reported as major degraders of macroalgal polysaccharides (52). *Saprospiraceae*, a predominant uncultured bacterial group in global WWTPs, are known for hydrolyzing and utilizing complex carbon molecules (53). The robust carbon-degrading capability (Fig. S5a) and higher abundance (Fig. S4a; Table S3) of these taxa in biofloc aquaculture ensure the efficient breakdown of complex organic matter, contributing to water quality maintenance and sustainable aquaculture. Our findings align with the established fact that *Bacteroidota* harbor numerous polysaccharide-degrading genes, often organized in specialized clusters known as polysaccharide utilization loci (PUL) (54). Marine *Bacteroidota* are particularly recognized for surface colonization and depolymerizing the complex organic matter (55).

Controlling ammonia, nitrite, and nitrate is critical in biofloc aquacultures (56), making nitrogen metabolism a focal concern. Bacteria in biofloc systems can support three ammonia-oxidizing processes: anammox, autotrophic nitrification, and HN-AD. Anammox, the anaerobic oxidation of ammonium and nitrite into nitrogen gas (57), was not evident in the FAB community (Fig. 4b; Table S11). The genomic potential for autotrophic nitrification, sequential oxidation of ammonia to nitrate via nitrite, was found to be minimal in the FAB community. This was also supported by the recovery of only six MAGs affiliated with well-known nitrifying groups, including ammonium-oxidizing bacteria (two MAGs of the genus *Nitrosomonas*) and nitrite-oxidizing bacteria (three MAGs of the phylum *Nitrospirota* and one *Nitrococcus* MAG) (Table S3). This finding corroborates with our recent study highlighting a minor proportion of nitrifiers in biofloc microbiota (4).

Considering the absence of anammox and minimal autotrophic nitrification, we explored the possibility of HN-AD. Complete nitrogen removal typically involves autotrophic nitrification under aerobic conditions, followed by heterotrophic denitrification under anoxic conditions (58). However, in HN-AD, both processes are performed aerobically by heterotrophs (59). While the understanding of this process is fragmentary, various studies have proposed the participation of two key enzymes: DnfABC, responsible for oxidizing ammonia to hydroxylamine and subsequent conversion of hydroxylamine to dinitrogen, and POD, which catalyzes the breakdown of pyruvic oxime to pyruvate and nitrate (36, 60). We investigated the presence of these marker proteins in our MAGs collection and found no genomic evidence for HN-AD in the FAB community (Fig. S6a and b).

While BFT principally relies on heterotrophic ammonium assimilation, the key heterotrophs involved remain unidentified. Ammonium assimilation is more effective for biological nitrogen removal than nitrification-denitrification in saline environments, as it alleviates nitrite/nitrate accumulation and greenhouse gas emissions (61). Previous studies have even developed a halophilic ammonium-assimilating microbiome for efficient nitrogen removal from saline wastewater (62). Our study confirms that heterotrophic assimilation is the main pathway responsible for controlling toxic ammonium in biofloc aquaculture. Dominant FAB community members, including *Flavobacteriaceae* (genera *Muricauda*, *Polaribacter*, and *Maribacter*), *Halieaceae* (*Halioglobus* and *Aequoribacter*), *Rhodobacteraceae* (*Marivita*, *Ruegeria*, *Donghicola*, and *Amaricoccus*), *Saprospiraceae* (*Phaeodactylibacter* and JABDMH01), and *Sphingomonadaceae (Alteraurantiacibacter*), are recognized as major ammonium assimilators utilizing the GS-GOGAT pathway (Fig. S7; Table S12). These taxa play a crucial role in efficiently removing ammonium, contributing to the sustainability and efficiency of biofloc systems. Maintaining and manipulating their abundance could optimize BFT for improved water quality and aquaculture productivity.

Denitrification and DNRA are major nitrogen metabolism pathways supported by the FAB community (Fig. 5a through c). Earlier studies have consistently reported nitrate accumulation in biofloc aquacultures (63), further supporting the possible occurrence of Denitrification and DNRA. Typically, these processes are confined to anoxic environments, including soil, sediment, deep-sea, and marine oxygen minimal zones (64). In fact, previous studies argued that bioflocs could not support denitrification due to the higher dissolved oxygen level (>5 mg L^−1^) in the peripheral water (35). Nevertheless, bioflocs range from size 50 to 1,000 µm and continuos microbial activities within large-sized bioflocs could create localized low-oxygen microenvironments (65). Moreover, continual accumulation of microbial extracellular polymeric may impede oxygen diffusion (66). Particle-attached microbial community can reduce oxygen permeability even in well-oxygenated bulk water, facilitating the growth of heterotrophic denitrifiers (67).

Recent studies have highlighted the existence of aerobic denitrifiers, contrasting with the traditionally understood anaerobic denitrifiers (68). Membrane-bound nitrate reductases (*narGHI*) are susceptible to oxygen and typically indicate anaerobic denitrification (69). Conversely, periplasmic nitrate reductases (*napAB*) expressed under both aerobic and anaerobic conditions, reliably indicate aerobic denitrification (70). In our investigation, MAGs affiliated with *Emcibacteraceae*, *Microbacteriaceae*, *and Rhodobacteraceae* exhibited complete *narGHI*, while members of *Flavobacteriaceae*, *Halieaceae*, *Rhodobacteraceae*, and *Planctomycetota*, harbored both *napAB* subunits, indicating the potential occurrence of both anaerobic and aerobic denitrifiers, respectively (Fig. 4a and b; Table S11). The coexistence of both aerobic and anaerobic denitrifiers has been frequently reported in biofloc aquaculture (71) and similar environments such as WWTPs (72). This dual presence offers promising prospects for nutrient cycling and water quality management. By efficiently converting nitrate into nitrogen gas, these microbes potentially play a crucial role in maintaining water quality. Their presence underscores the potential for biofloc systems to function as self-regulating and sustainable ecosystems, effectively recycling nitrogenous compounds and minimizing environmental impacts.

Aquaculture systems, especially those operating based on BFT, receive higher organic inputs than other natural aquatic environments. The microbial decomposition of these diverse organic matters leads to significant hydrogen sulfide production, a toxic gas that can cause severe economic losses in the aquaculture industry (73). Therefore, biological removal of hydrogen sulfide through sulfur-oxidizing bacteria is considered an effective and sustainable strategy (74). Previous study successfully constructed a sulfide-oxidizing microbiota using halophilic microorganisms for the biological remediation of sulfide-rich aquaculture environment (75).

To address the necessity to convert sulfide into less toxic forms, such as sulfate, we examined the sulfur-oxidizing potential of the FAB community. Several reconstructed MAGs harbored a full suite of *soxXAYZBCD* genes (Fig. 5a and c; Table S14), potentially enabling the oxidation of various reduced sulfur compounds, including sulfide, sulfite, and thiosulfate to sulfate. This suggests that a significant portion of the FAB community is involved in sulfur oxidation cycling, contributing to water quality maintenance. Intriguingly, these MAGs were predominantly affiliated with *Rhodobacteraceae*, a group renowned for the degradation and oxidation of sulfur compounds in marine environments (47). *Rhodobacteraceae* may, therefore, play a crucial role in controlling sulfide levels in aquaculture settings. Similar to the FAB community, *Rhodobacteraceae* members have been reported to participate in nitrate-dependent oxic/anoxic sulfide oxidation in recirculating aquaculture systems (76). Furthermore, a recent study highlighted their abundance and capability for thiosulfate oxidation under both aerobic and anaerobic conditions in natural marine biofilms (66).

Some MAGs affiliated with the order *Arenicellales* (formerly UBA10353) also possessed *rdsr*-mediated sulfur oxidation potential (Fig. 5b; Table S15). This pathway reverses the sulfate reduction process found in sulfate-reducing bacteria, oxidizing sulfide to sulfate through sulfite (77). The co-occurrence of both *sox*-mediated and *rdsr*-mediated pathways implies the versatile sulfur metabolic potential of this bacterial taxon. These bacteria could execute sulfur oxidation in two ways: (i) direct thiosulfate oxidation to sulfate using *sox* gene cluster, and (ii) initial oxidation of thiosulfate or sulfide to elemental sulfur, followed by further oxidation to sulfate via the *rdsr*-mediated pathway (78). Supporting our findings, the UBA868 family within the order *Arenicellales* has recently been identified as ubiquitous sulfur oxidizers across the global mesopelagic ocean, utilizing both *sox*-mediated and *rdsr*-mediated pathways (79).

### Conclusion

Genomic analysis sheds lights on the metabolic capabilities of the biofloc microbiota, highlighting their potential for both aerobic and anaerobic metabolisms. Key metabolic pathways identified include ammonium assimilation, denitrification, dissimilatory nitrate reduction to ammonia, thiosulfate oxidation, and sulfide oxidation. Though present in minor abundance, autotrophic nitrification is the primary pathway for ammonium oxidation in biofloc aquacultures, with genomic evidence not supporting anammox and heterotrophic nitrification. More importantly, this study reinforces the notion that heterotrophic ammonium assimilation is the primary pathway for managing toxic ammonium levels in biofloc aquaculture, with a diverse array of heterotrophic bacterial taxa playing a key role in this process. The presence of microhabitats, possibly within large-sized bioflocs or due to the accumulation of microbial extracellular polymeric substances, could support anaerobic processes such as denitrification and sulfide oxidation despite the overall well-oxygenated conditions of the rearing water. Members of the *Rhodobacteraceae* were identified as the most dominant, core, and metabolically versatile taxa in the biofloc aquaculture. This bacterial taxon serves as major probiont and is potentially involved in several crucial ecological functions, including carbohydrate degradation, ammonium assimilation, denitrification, and sulfide/thiosulfate oxidation.

## SUPPLEMENTAL MATERIAL

Supplemental Figures msystems.00782-24-s0001.docx Figures S1-S8.

Supplemental Tables msystems.00782-24-s0002.xlsx Tables S1-S15.

ASM does not own the copyrights to Supplemental Material that may be linked to, or accessed through, an article. The authors have granted ASM a non-exclusive, world-wide license to publish the Supplemental Material files. Please contact the corresponding author directly for reuse.
