# Supplementary material for: Genome-centric metagenomics provides insights into the core microbial community and functional profiles of biofloc aquaculture
Source: mSystems. 2024 Sep 24;9(10):e00782-24. doi: 10.1128/msystems.00782-24 (PMC11494986; doi:10.1128/msystems.00782-24)
Supplement: Supplemental Figures — Figures S1-S8. [file msystems.00782-24-s0001.docx]

**Supplementary material for manuscript entitled “Genome-centric Metagenomics Provides Insights into the Core Microbial Community and Functional Profiles of Biofloc Aquaculture”**

Meora Rajeev^a,b^, Ilsuk Jung^a^, Ilnam Kang^c^, and Jang-Cheon Cho^a,c*^

^a^Department of Biological Sciences and Bioengineering, Inha University, Incheon, Republic of Korea

^b^Institute for Specialized Teaching and Research, Inha University, Incheon, Republic of Korea

^c^Center for Molecular and Cell Biology, Inha University, Incheon, Republic of Korea

Running Head**:** Metagenomic profiling of biofloc microbiota

*Address correspondence to Jang-Cheon Cho, e-mail address: [chojc@inha.ac.kr](mailto:chojc@inha.ac.kr)


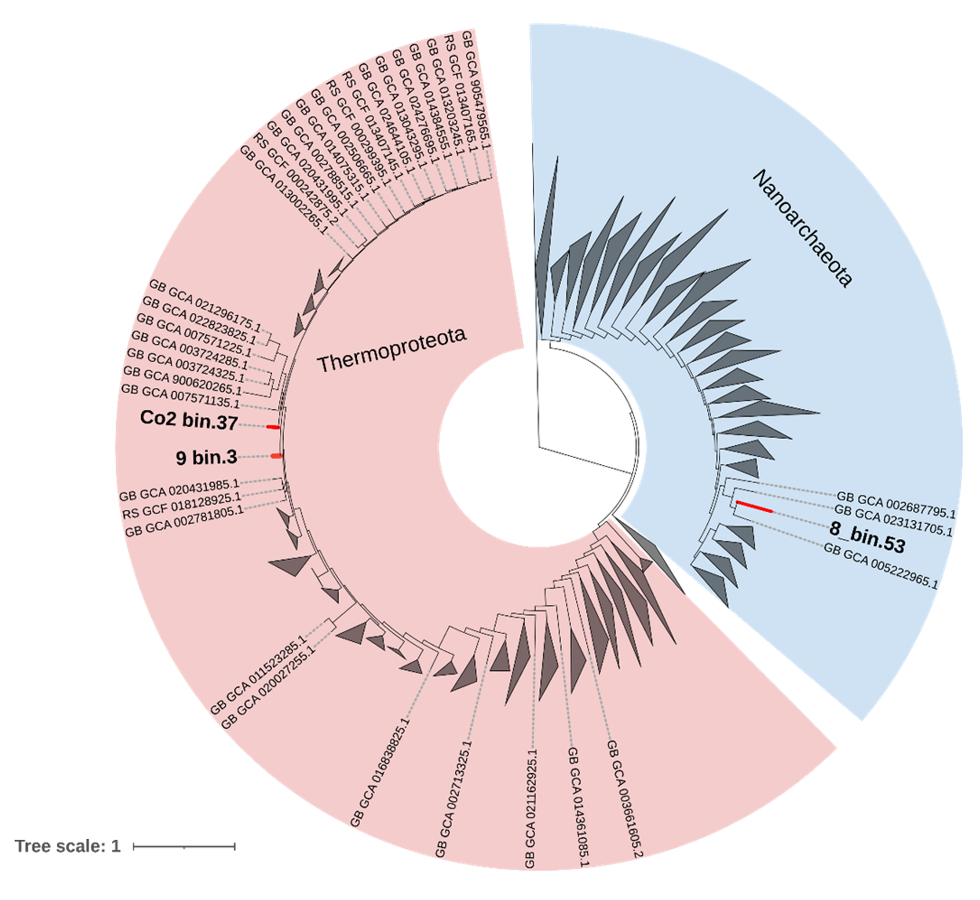


**Figure S1.** Phylogenetic affiliations of archaeal MAGs. The phylogenetic tree shows phylogenetic positions of three archaeal MAGs recovered from the floc-associated microbial (FAB) community. The tree was constructed using FastTree program (options: -lg -gamma) based on 53 archaeal-specific single-copy marker proteins alignments by the GTDB-Tk. Detailed taxonomy of three archaeal MAGs is provided in Table S4.


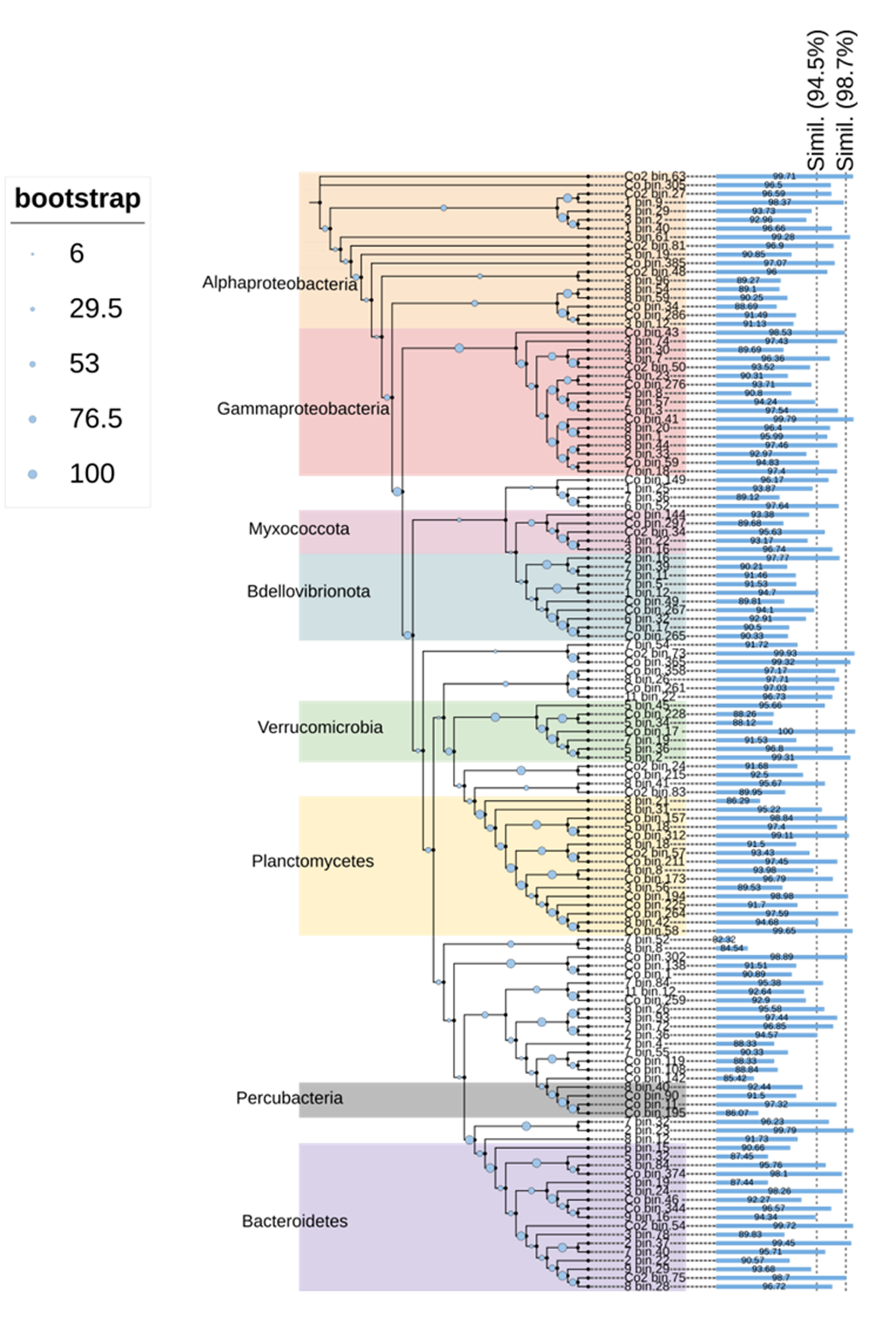


**Figure S2.** 16S rRNA gene phylogenetic tree of floc-associated bacterial (FAB) community. This figure depicts a maximum-likelihood phylogenetic tree constructed using the 16S rRNA gene sequences extracted from the metagenome-assembled genomes (MAGs). The bar plots illustrate the similarity of these sequences in the EZBioCloud database. The vertical lines delineate thresholds for defining novel bacterial genera (94.5% similarity) and species (< 98.7% similarity). Detailed taxonomy is provided in Table S5. The tree was built using RAxML with 1000 bootstraps.


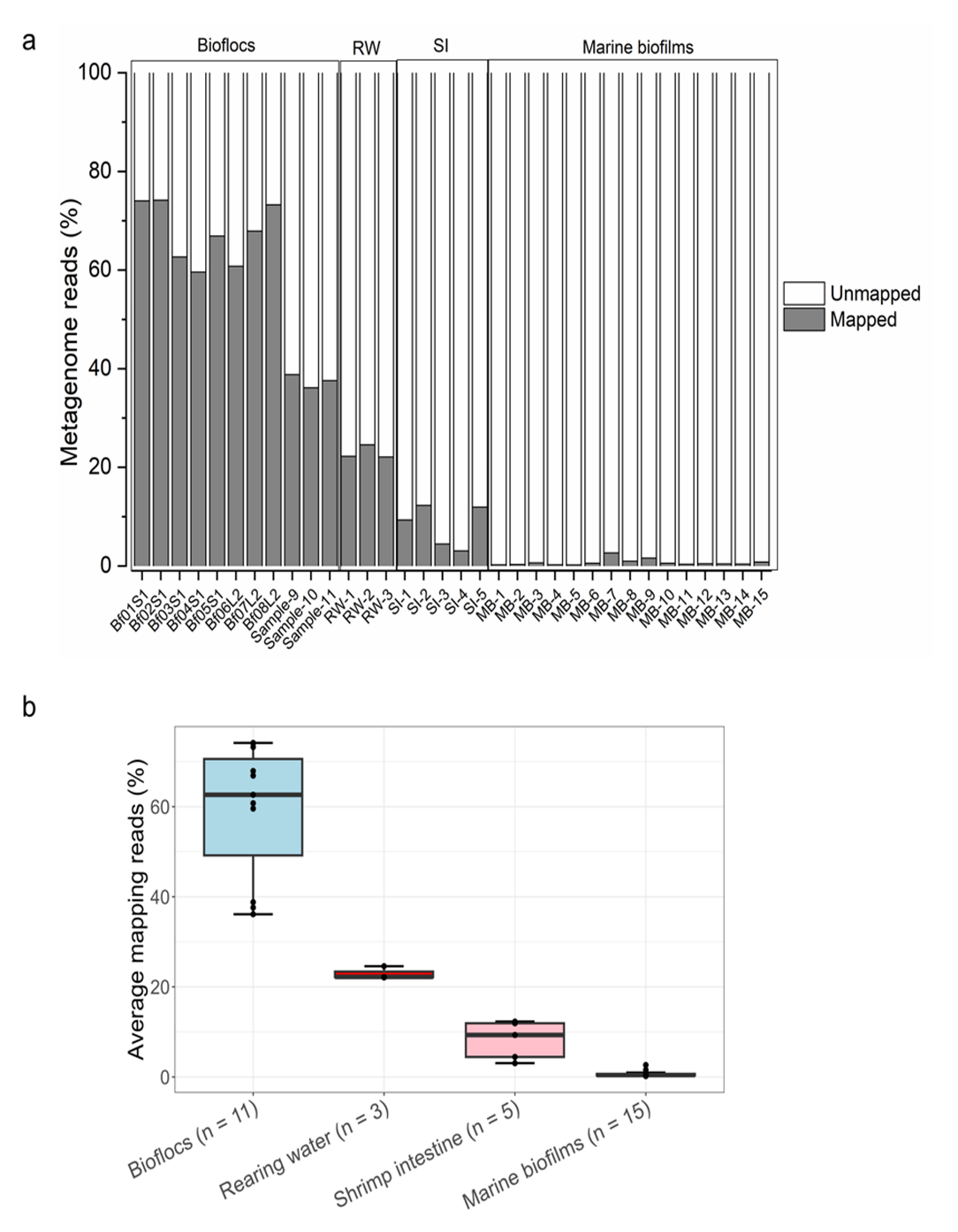


**Figure S3.** Coverage of MAGs compendium. (**a**) Bar plots represent metagenomic reads mapping rates of bioflocs and other similar environments on recovered MAGs collection in this study. (**b**) Box plots illustrate average reads mapping on various metagenomes. Sample-specific details of reads mapping for each investigated metagenome are provided in Table S7. Metagenomes from rearing water, shrimp intestines, and marine biofilms, as reported in previous studies (1–3), were downloaded from the SRA database.


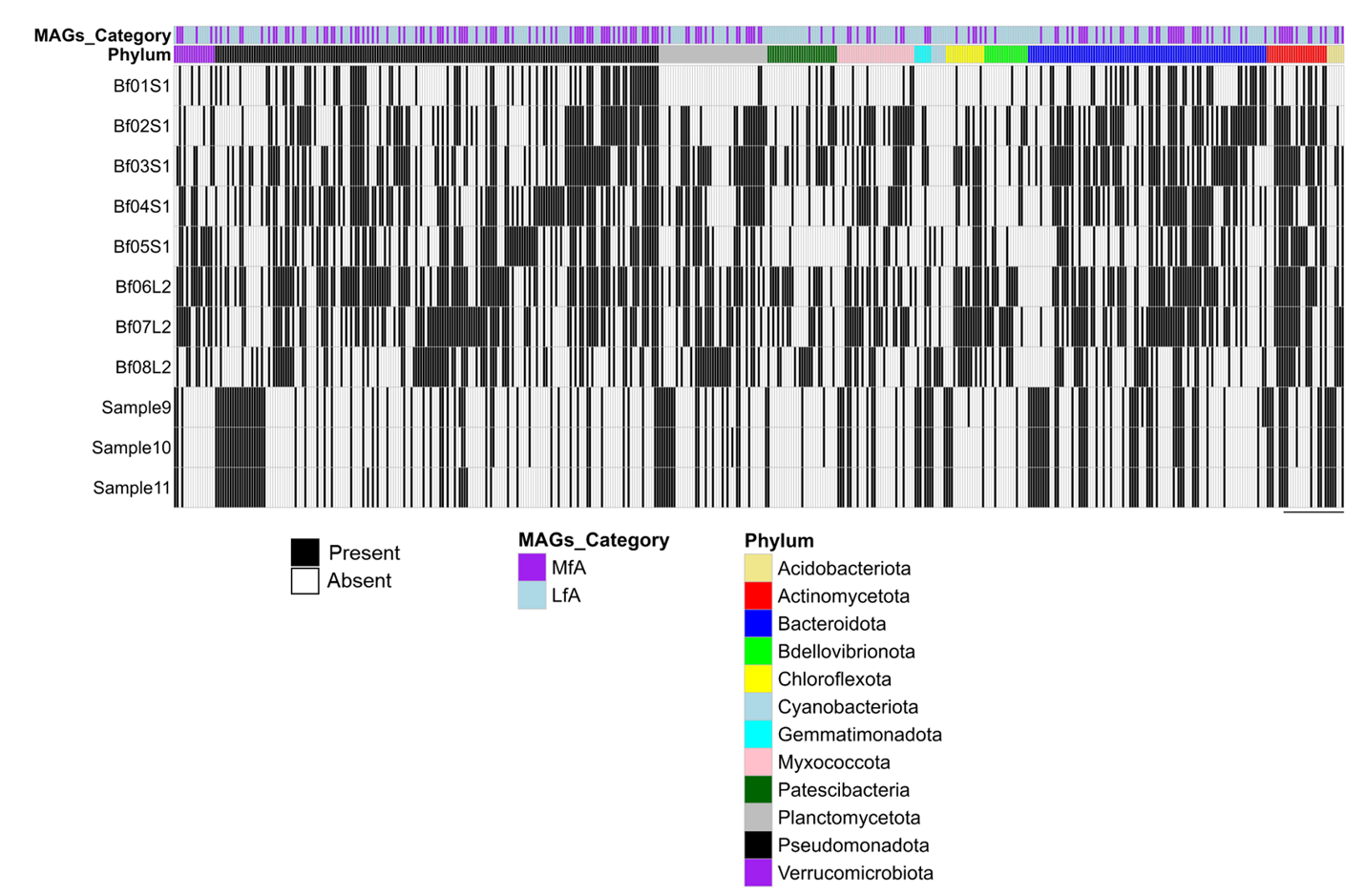


**Figure S4.** Distribution of recovered bacterial metagenome-assembled genomes (MAGs) across investigated biofloc metagenomes. The heatmap depicts the presence/absence patterns of MAGs in biofloc metagenomes, and their corresponding taxonomic classification at the phylum level. MAGs were categorized as most frequently appeared (MfA), if present in ≥ 6 samples, or least frequently appeared (LfA), if present in ≤ 5 samples. The determination of MAGs appearance was based on reads mapping of biofloc metagenomes onto MAGs using CoverM. Three metagenomes (Sample9–Sample11) were downloaded from an earlier study (3). Detailed relative abundances of each MAG across investigated metagenomes and their categorization are provided in Table S8.


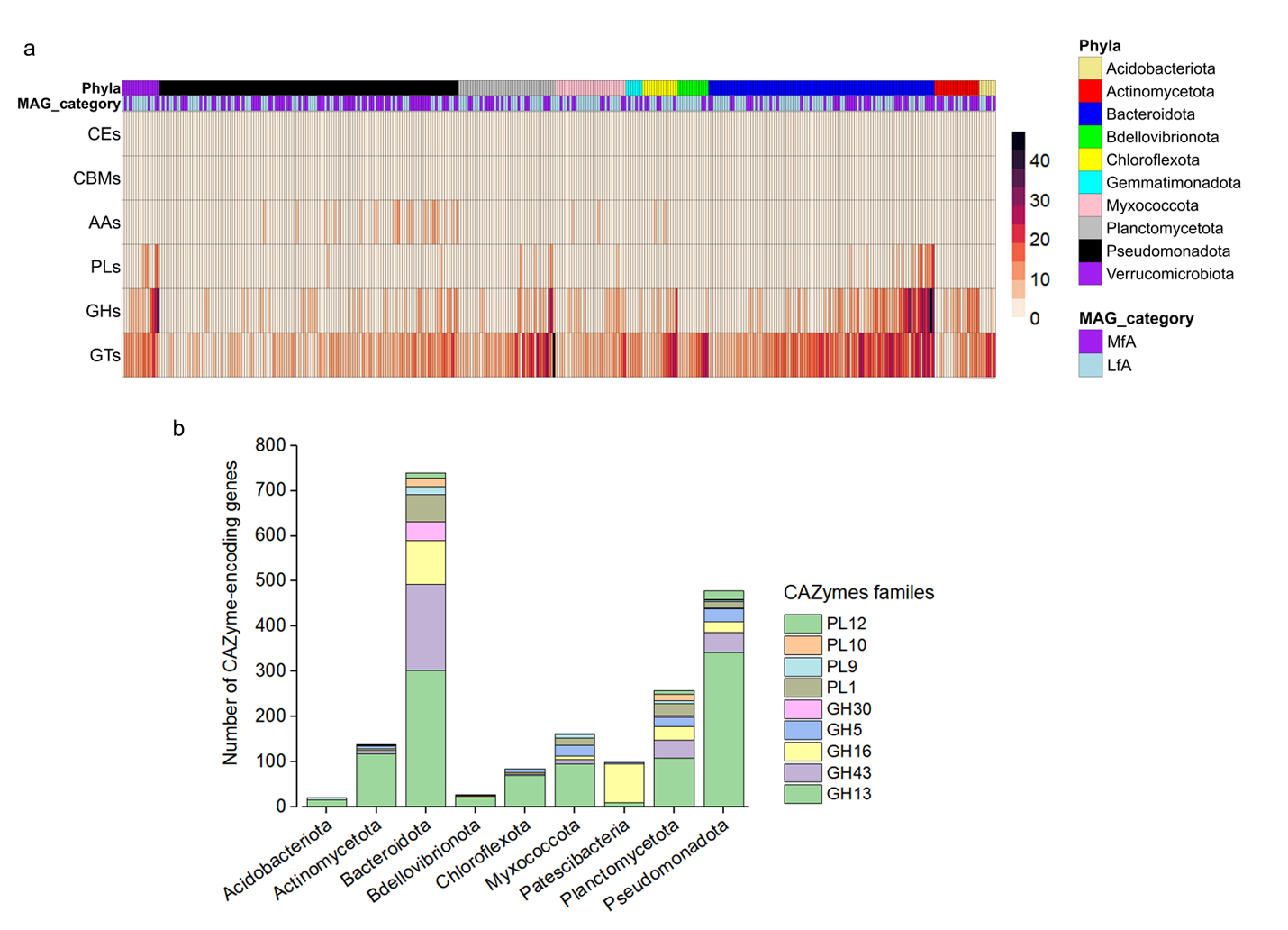


**Figure S5.** Carbohydrate utilization potential of the floc-associated microbial (FAB) community. (**a**) Heatmap illustrating the distribution of carbohydrate-active enzymes (CAZymes) encoded by the bacterial MAGs across six major classes. (**b**) Stacked bar plot displaying the major bacterial phyla of the FAB community and the CAZymes families they encode for. Abbreviations: GTs, glycosyltransferases; GH, glycoside hydrolases; AAs, auxiliary activities; PLs, polysaccharide lyases; CEs, carbohydrate esterases; CBMs, carbohydrate-binding modules. Detailed information pertaining to CAZymes abundance in the FAB community is provided in Table S10.


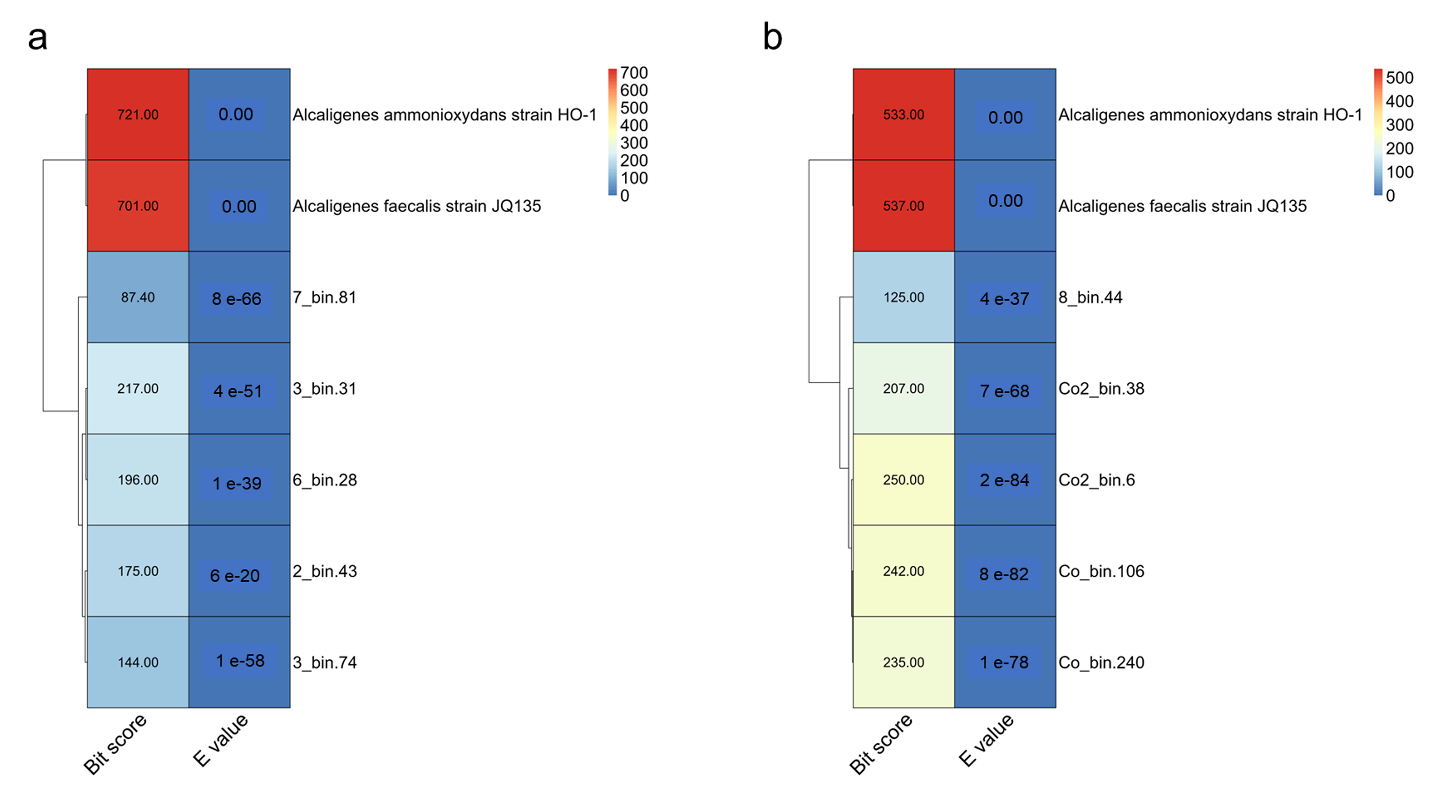


**Figure S6.** Evidence for the absence of heterotrophic nitrifiers in biofloc aquaculture. Heatmaps represent the result of BLASTp analyses for two enzymes, namely (**a**) dinitrogen-forming (DnfABC, GenBank protein identifiers QXX79842–QXX79844), and (**b**) pyruvic oxide dioxygenase (POD, GenBank protein identifier ASR28422) against two reference bacterial strains reported earlier (4, 5) as well as MAGs recovered in this study. These enzymes served as functional markers for the heterotrophic nitrification – aerobic denitrification (HN-AD) process. Only the top 5 MAGs showing the highest similarities are represented. Values on the columns represent bit score similarities and E-values for the respective genomes.


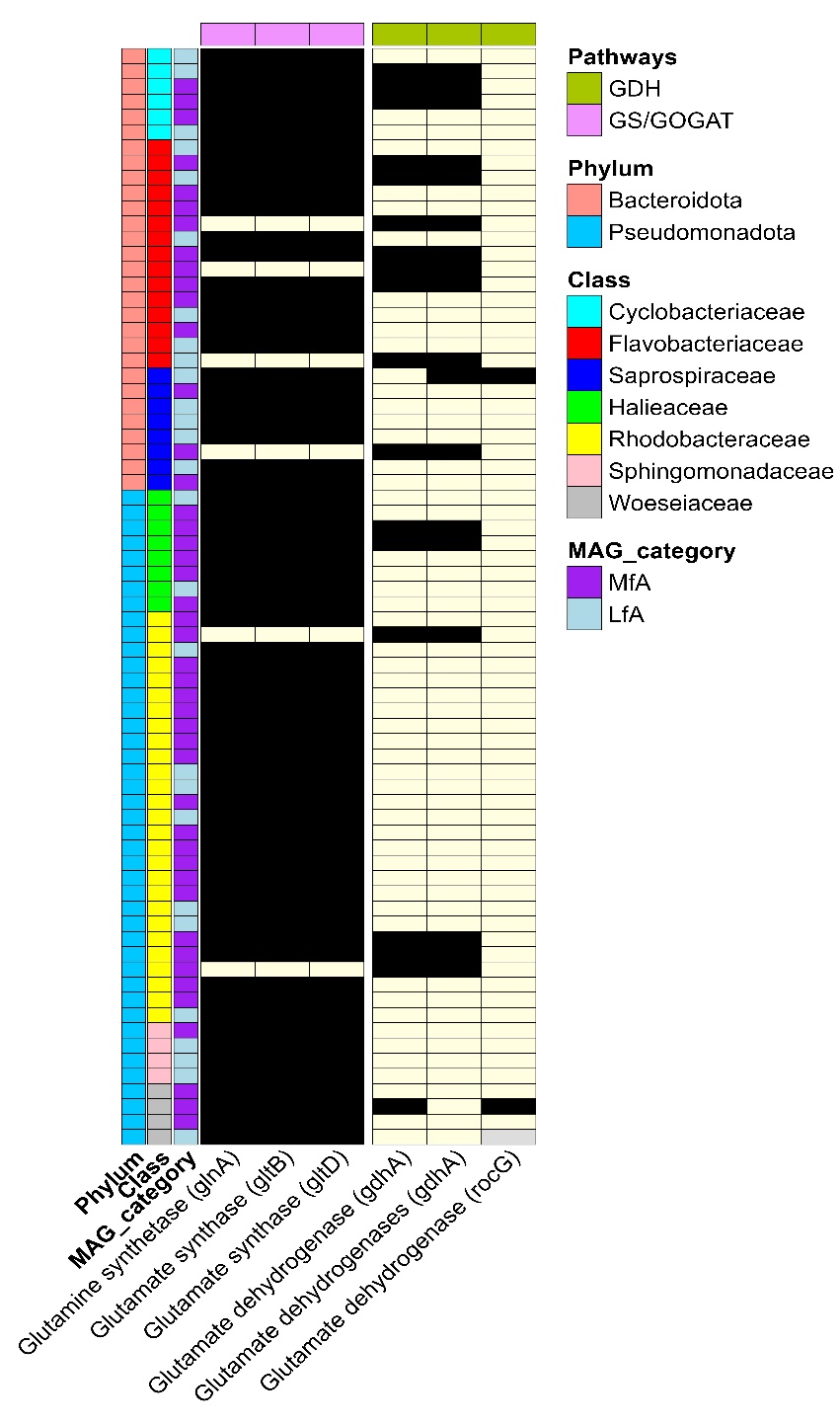


**Figure S7.** Ammonium assimilation potential of the floc-associated microbial (FAB) community. The heatmap represents the MAGs harboring genes involved in two pathways for ammonium assimilation: glutamine synthetase/glutamate synthase (GS/GOGAT) and glutamate dehydrogenase (GDH). Only major bacterial taxonomic groups (represented by ≥ 3 MAGs) are represented in this figure. The entire collection of recovered MAGs potentially involved in ammonium assimilation is listed in Table S12. Bacterial MAGs possessing glutamine synthetase (*glnA*, K01915) and glutamate synthases (*gltB*, K00265; *gltD*, K00266) were considered to be involved in the GS/GOGAT pathway, while MAGs possessing glutamate dehydrogenases (*gdhA*, K00261; *gdhA*, K00262; *rocG*, K15371) were considered for the GDH pathway.


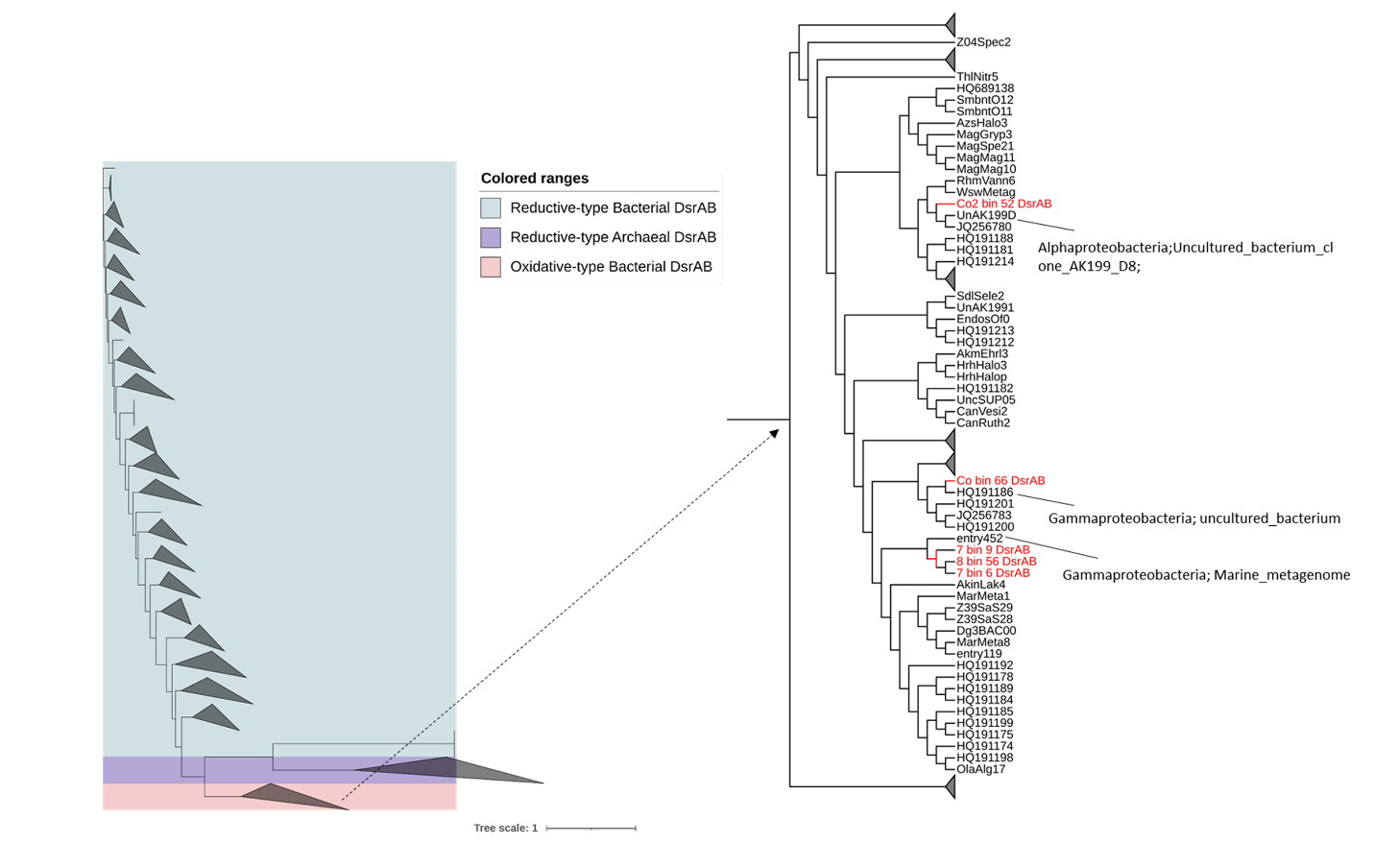


**Figure S8.** Phylogenetic positioning of reverse/oxidative dissimilatory sulfite reductase (*rdsrAB*) genes found in the five bacterial MAGs recovered from the floc-associated bacterial (FAB) community. The reference *dsrAB* genes file was downloaded from a previous study (6), and multiple sequence alignment with the *dsrAB* genes obtained in this study was conducted using the MAFFT program. The five MAGs recovered in this study are highlighted in red font. The phylogenetic tree was constructed using FastTree with options -lg -gamma, and the annotation was performed using Interactive Tree of Life (iTOL) v6.8.

**References**

1. Gao S, Pan L, Huang F, Song M, Tian C, Zhang M. 2019. Metagenomic insights into the structure and function of intestinal microbiota of the farmed Pacific white shrimp (*Litopenaeus vannamei*). Aquaculture 499:109–118.
2. Zhang W, Ding W, Li YX, Tam C, Bougouffa S, Wang R, Pei B, Chiang H, Leung P, Lu Y, Sun J, Fu H, Bajic VB, Liu H, Webster NS, Qian PY. 2019. Marine biofilms constitute a bank of hidden microbial diversity and functional potential. Nat Commun 10:517.
3. Chen X, He Z, Zhao J, Liao M, Xue Y, Zhou J, Hoare R, Monaghan SJ, Wang N, Pang H, Sun C. 2022. Metagenomic analysis of bacterial communities and antibiotic resistance genes in *Penaeus monodon* biofloc-based aquaculture environments. Front Mar Sci 8:762345.
4. Wu MR, Hou TT, Liu Y, Miao LL, Ai GM, Ma L, Zhu HZ, Zhu YX, Gao XY, Herbold CW, Wagner M, Li DF, Liu ZP, Liu SJ. 2021. Novel *Alcaligenes ammonioxydans* sp. nov. from wastewater treatment sludge oxidizes ammonia to N_2_ with a previously unknown pathway. Environ Microbiol 23:6965–6980.
5. Mu Y, Xu S, Liu G, Cheng M, Dai W, Chen Q, Yan X, Hong Q, He J, Jiang J, Qiu J. 2022. The novel monooxygenase gene *dipD* in the dip gene cluster of *Alcaligenes faecalis* JQ135 is essential for the initial catabolism of dipicolinic acid. Appl Environ Microbiol 88:e00360-22.
6. Pelikan C, Herbold CW, Hausmann B, Müller AL, Pester M, Loy A. 2016. Diversity analysis of sulfite‐and sulfate‐reducing microorganisms by multiplex *dsrA* and *dsrB* amplicon sequencing using new primers and mock community‐optimized bioinformatics. Environ Microbiol 18:2994–3009.
